# Evaluation of a phosphate kinetics model in hemodialysis therapy—Assessment of the temporal robustness of model predictions

**DOI:** 10.14814/phy2.15899

**Published:** 2023-12-21

**Authors:** Sisse H. Laursen, Lise Boel, Lisbet Brandi, Jeppe H. Christensen, Peter Vestergaard, Ole Kristian Hejlesen

**Affiliations:** ^1^ The Danish Diabetes Academy Odense University Hospital Odense Denmark; ^2^ Department of Health Science and Technology Aalborg University Aalborg Denmark; ^3^ Department of Nursing University College of Northern Denmark Aalborg Denmark; ^4^ Steno Diabetes Center North Jutland Aalborg University Hospital Aalborg Denmark; ^5^ Clinical Nursing Research Unit Aalborg University Hospital Aalborg Denmark; ^6^ Department of Clinical Medicine Aalborg University Aalborg Denmark; ^7^ Department of Cardiology, Nephrology, and Endocrinology, Nordsjællands Hospital Hillerød Denmark; ^8^ Department of Nephrology Aalborg University Hospital Aalborg Denmark; ^9^ Department of Endocrinology Aalborg University Hospital Aalborg Denmark

**Keywords:** compartment modeling, dialysis, hyperphosphatemia, kinetics, phosphate

## Abstract

In‐depth understanding of intra‐ and postdialytic phosphate kinetics is important to adjust treatment regimens in hemodialysis. We aimed to modify and validate a three‐compartment phosphate kinetic model to individual patient data and assess the temporal robustness. Intradialytic phosphate samples were collected from the plasma and dialysate of 12 patients during two treatments (HD1 and HD2). 2‐h postdialytic plasma samples were collected in four of the patients. First, the model was fitted to HD1 samples from each patient to estimate the mass transfer coefficients. Second, the best fitted model in each patient case was validated on HD2 samples. The best model fits were determined from the coefficient of determination (*R* ^2^) values. When fitted to intradialytic samples only, the median (interquartile range) *R* ^2^ values were 0.985 (0.959–0.997) and 0.992 (0.984–0.994) for HD1 and HD2, respectively. When fitted to both intra‐ and postdialytic samples, the results were 0.882 (0.838–0.929) and 0.963 (0.951–0.976) for HD1 and HD2, respectively. Eight patients demonstrated a higher *R* ^2^ value for HD2 than for HD1. The model seems promising to predict individual plasma phosphate in hemodialysis patients. The results also show good temporal robustness of the model. Further modifications and validation on a larger sample are needed.

## 1 INTRODUCTION

Hyperphosphatemia (plasma phosphate >1.4 mmol/L) (Wheeler & Winkelmayer, 2017) constitutes one of the most common and challenging conditions in hemodialysis (HD) therapy affecting approximately 50%–80% of the HD population (Barreto et al., 2019; Fouque et al., 2018; Laursen et al., 2017; Vikrant & Parashar, 2016). The condition is mainly caused by the reduced urinary excretion of phosphate due to the impaired renal function. Thus, the impaired renal function causes an accumulation of phosphate as the excretion of phosphate no longer matches the absorption of phosphorus from the gastrointestinal tract (Eknoyan et al., 2013; Nadkarni & Uribarri, 2014; Wheeler & Winkelmayer, 2017). Furthermore, various hormones such as parathyroid hormone (PTH) and fibroblast growth factor 23 (FGF‐23) play a key role. Normally, both hormones induce increased urinary excretion of phosphate, and PTH also leads to skeletal phosphate resorption, while FGF‐23 decreases renal phosphate reabsorption (Moe, 2010; Nadkarni & Uribarri, 2014; Perwad et al., 2007; Wheeler & Winkelmayer, 2017). However, in this advanced stage of kidney disease (stage 5), these hormones do not longer compensate sufficiently for the continuous input of phosphate from fluid and dietary intake leading to a positive phosphate balance (Galvao et al., 2013; Nadkarni & Uribarri, 2014). Furthermore, the use of vitamin D and other medications increases the intestinal absorption of phosphate, which aggravates the positive balance even further (Akimbekov et al., 2022). Hyperphosphatemia is associated with severe long‐term consequences in HD patients. For instance, the condition is known to accelerate vascular calcification processes mainly due to an increase in the calcium‐phosphate product (Cozzolino et al., 2001; Moe, 2010). Vascular calcifications are common in HD patients affecting approximately 70%–80% of the patient population (Moe, 2010) and are highly associated with cardiovascular events and premature death (Barreto et al., 2019; Mizobuchi et al., 2009; Moe, 2010). Thus, the risk of cardiovascular death is approximately 10–30 times higher in HD patients than in the general population (Ford et al., 2010; London, 2018). Another consequence of hyperphosphatemia in HD patients is hyperparathyroidism. Hyperparathyroidism can cause a wide range of symptoms including bone and joint pain, limb deformities, fragile bones kidney stones, nausea, loss of appetite, fatigue, immune system effects, etc. (Goodman, 2004; Llach & Forero, 2001; Martin et al., 2010). Another common consequence is renal osteodystrophy which causes a number of underlying bone diseases such as adynamic bone disorder, osteoporosis, osteitis fibrosa, and osteomalacia; diseases that may contribute to bone pain and fractures, myopathy, periarthritis, muscle pain, and tendon ruptures (Moe et al., 2006; Tan et al., 2018). Thus, the consequences linked to hyperphosphataemia impose a significant burden on both HD patients and healthcare resources. Therefore, control of this marker is crucial.

The management of hyperphosphatemia is currently based on four main strategies: (1) dietary phosphate restriction; (2) reduction of intestinal phosphorus absorption; (3) dialysis phosphate removal; and (4) prevention and treatment of renal osteodystrophy (Barreto et al., 2019; Kuhlmann, 2006). The persistent problem with hyperphosphatemia in dialysis patients indicates the need to improve the current interventions. In this study, we focused on phosphate removal in HD patients.

In‐depth understanding of individual intra‐ and postdialytic phosphate kinetics is a prerequisite to improve dialysis phosphate removal in HD patients. Kinetic modeling is a well‐known approach to study and understand physiological systems and processes. Hence, kinetic modeling may be a useful approach to better understand the individual's phosphate balance (Cobelli & Carson, 2019; Debowska et al., 2015; Farrell, 1983; Laursen et al., 2017). According to previous modeling studies (Agar et al., 2011; Debowska et al., 2015; Heaf et al., 1998; Heaf & Jensen, 1994; Laursen et al., 2015; Leypoldt et al., 2012; Maasrani et al., 1995; Pogglitsch et al., 1989; Ruggeri et al., 1997; Spalding et al., 2002; Sugisaki et al., 1983), there seems to be an agreement that a phosphate kinetics model should consist of ≥2 compartments to ensure the well‐documented stabilization of phosphate during HD (Desoi & Umans, 1993; Pogglitsch et al., 1984; Sugisaki et al., 1982). In four of these studies it is even indicated that more than two compartments are needed to describe the intradialytic phosphate rebound (Heaf et al., 1998; Laursen et al., 2015; Maasrani et al., 1995; Spalding et al., 2002). According to these modeling results, a two‐compartment model may be too simple to simulate intra‐ and postdialytic phosphate kinetics. However, eventhough phosphate kinetics models exist, no model seems to have gained clinical acceptance. This might be due to the fact that the models need further validation or that they are too complex to be used in clinical practice (Laursen et al., 2015).

We recently presented some promising two‐ and three‐compartment models describing intra‐ and postdialytic phosphate kinetics (Laursen et al., 2015). The model approach used in the present study is advantageous because the mathematical description is rather simple and considered easily accessible (Laursen et al., 2017). However, although promising, the model approaches are rather general and descriptive; they may be challenged by not describing the individual's phosphate kinetics and may not be entirely consistent with physiological processes. In this study, the aim was to modify the most promising model—a three‐compartment model—to make it agree more with physiological systems and to fit the model to individual intra‐ and postdialytic phosphate kinetics. To validate the model, it was first modified and validated for each patient individually based on data from one HD treatment. Without performing a new model fit, it was then tested on HD treatment data from the same patient 1 week later to assess the temporal robustness of model predictions.

## 2 MATERIALS AND METHODS

The data were collected in the spring of 2019 after approval from The National Ethics Committee in Denmark (Ethics journal number N‐20160088). All the patients provided written informed consent before the trial.

### 2.1 Patients and settings

Thirteen chronic HD patients (eight males and five females) undergoing dialysis at a hospital ward in Denmark were included in the study. Patients who were unstable (medically or psychologically), pregnant women or patients with a hemoglobin level lower than 6 mmol/L were not considered suitable for inclusion. Additionally, patients with unstable vascular access and those who were not stable on dialysis for at least 3 months were excluded. One of the participants was excluded due to technical issues, leaving 12 HD patients in the study. All the study subjects were Caucasian, and their median age was 70.5 (57–89) years. Eight of the patients were dialyzed using an arteriovenous fistula, and four patients used a catheter for vascular access. The patients had undergone maintenance HD for a median of 48 (6–144) months before the study began. The demographic characteristics are summarized in Table 1.

**TABLE 1:** Demographic characteristics (*n* = 12).

Characteristics	
Female, *n* (%)	4 (33.3)
Male, *n* (%)	8 (66.7)
Age (years), mean ± SD	71.6 ± 10.6
Dry weight (kg), mean ± SD	72.2 ± 14.2
Height (cm), mean ± SD	160.3 ± 8.2
Body mass index (kg/m^2^), mean ± SD	23.9 ± 4.3
Total body water (l), mean ± SD	39.3 ± 6.5
Duration of HD (months), mean ± SD	53.5 ± 46.2
Cause of kidney disease	
Kidney cysts, *n* (%)	2 (16.7)
Infection (urinary tract), *n* (%)	1 (8.3)
Hypertension, *n* (%)	4 (33.3)
Kidney stones, *n* (%)	2 (16.7)
Unknown or other, *n* (%)	3 (25.0)

Abbreviations: HD, hemodialysis, SD, standard deviation.

### 2.2 Dialysis and sampling techniques

Dialysis was performed using a Fresenius dialysis machine (5008 or 6008) and high‐flux dialyzers (FX 60, FX 80, or FX 100). The dialyzer type was the same for each patient during HD1 and HD2. The blood flow rates were 230 to 350 mL/min, and the dialysate flow rates were 318 to 547 mL/min.

Each patient was studied during two separate HD treatments (231.3 ± 25.8 min), but on the same day (mid‐dialysis) in the week. A predialytic blood sample was collected before each session from the vascular access. Additional samples were drawn from the arterial blood tubing of the extracorporeal circuit. The intradialytic blood samples were collected each half hour and at the end of treatment. In addition to intradialytic blood samples, postdialytic blood samples were collected from four of the patients 30, 60, 90, and 120 min after dialysis completion to determine postdialytic phosphate kinetics (all the patients were asked whether they would participate in this part of the study, and four patients agreed). Moreover, the dialysate samples from the dialysate outflow were collected from all 12 patients each hour, from the 1‐h time point to the end of the treatment, to determine the dialyzer phosphate clearance. All the blood and dialysate samples were analyzed for the phosphate concentration.

Changes in the treatment parameters during HD were recorded, and relevant information was obtained from the patient's file or by asking the patient directly. This included information about relevant medication, dialysis prescriptions, kidney disease, dialysis history, comorbidities, age, gender, body weight (predialysis), and height. The study subjects abstained from diet, fluid, and medication intake during the trial to prevent influencing factors from affecting the blood phosphate concentration.

### 2.3 Analytical assays

According to the local guidelines, all the blood samples were allowed to stand at room temperature for up to 12 h without any additional anticoagulant or preservatives. The dialysate samples were stored at −80°C until assayed. All the samples were analyzed using an automated analyzer (COBAS 8000; module c702). In a few cases, the dialysate phosphate concentration was immeasurable; these samples were not included in the calculation of the dialyzer clearance. Additionally, low‐value (<0.10 mmol/L) samples were considered outliers. During nine of the HD treatments, two separate dialysate samples were collected at each dialysate sampling time (*n* = 2×9×4) 2 to test whether the automated analyzer provided reliable test results because the dialysate samples were not routinely assayed at the hospital. The median coefficient of variance value of the dual samples was 5.6%, indicating a relatively high test precision.

### 2.4 Kinetic model

**FIGURE 1:**
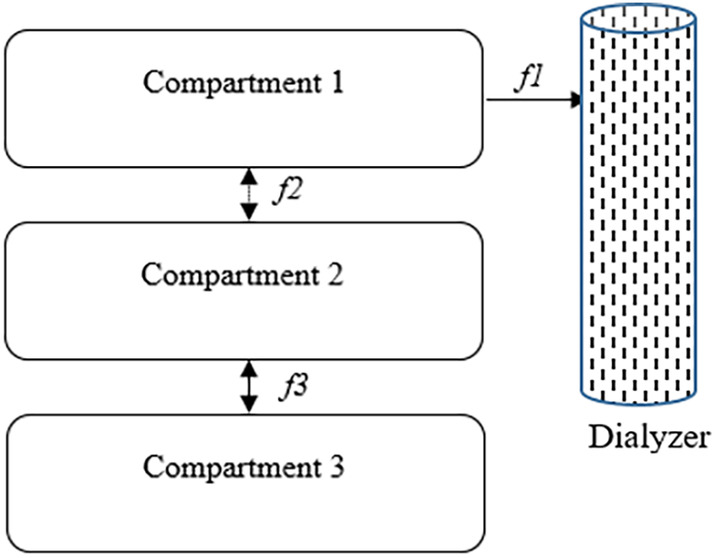
Structure of the three‐compartment model. The component f1 is the transport component of phosphate between dialysate and compartment 1 during dialysis, and f2 and f3 are diffusive transport components of phosphate between compartments (mmol/min).

The model builds on a three‐compartment model previously presented as model variation numbers 8 and 10 (fitted to four‐ and eight‐hour HD, respectively) as described by Laursen et al. (Laursen et al., 2015). The components of the model are presented in Table 2, and the model structure is illustrated in Figure 1. In this study, modifications were made to the volumes of distribution in the three compartments (V_1_, V_2_, and V_3_), dialyzer phosphate clearance (k_d_) and two mass transfer coefficients (k_1_ and k_2_). The components V_1_, V_2_, V_3_, and k_d_ were calculated and remained fixed for each patient, whereas k_1_ and k_2_ were estimated. The calculation and estimation of the components are available in the subsections below. Furthermore, the components and equations of the modified model are outlined in the Data S1, and the model is publicly available through Zenodo (https://zenodo.org/records/10052961).

**TABLE 2:** Components of the three‐compartment model.

Component	Description	Unit
*f*_*1*_	Phosphate eliminated through dialysis clearance	mmol/min
*f*_*2*_	Phosphate diffused between compartment 1 and 2	mmol/min
*f*_*3*_	Phosphate diffused between compartment 2 and 3	mmol/min
M_1_	The mass of phosphate in compartment 1	mmol
M_2_	The mass of phosphate in compartment 2	mmol
M_3_	The mass of phosphate in compartment 3	mmol
C_1_	Concentration of phosphate in compartment 1	mmol/l
C_2_	Concentration of phosphate in compartment 2	mmol/l
C_3_	Concentration of phosphate in compartment 3	mmol/l
C_d_	Concentration of phosphate in the dialysate	mmol/l
V_1_	Volume of distribution in compartment 1	l
V_2_	Volume of distribution in compartment 2	l
V_3_	Volume of distribution in compartment 3	l
*k*_*d*_	Dialyzer clearance of phosphate	l/h
*k*_*1*_	Mass transfer coefficient 1	l/h
*k*_*2*_	Mass transfer coefficient 2	l/h
*s*	Dialysis status	(0 = no, 1 = yes)

#### 2.4.1 Model implementation

Modeling was performed using Microsoft Office Excel 2013, and the measured plasma phosphate values were applied to modify and validate the model. First, the measured plasma phosphate values, as well as the average dialyzer clearance, from HD1 was used to modify and validate the model for each patient individually. Second, the measured plasma phosphate values from HD2 were used to test the model from HD1 in each patient case without changing any model component.

#### 2.4.2 Determination of volumes of distribution

The volume of distribution in compartment 1 (V_1_) was assumed to be equal to the fluid in plasma. The volume of distribution in compartment 2 (V_2_) was assumed to be the remaining fluid in the extracellular fluid (ECF) from the expection that V_2_ = ECF − V_1_. The volume of distribution in compartment 3 (V_3_) was assumed to be the intracellular fluid and thus equal to total body water (TBW) minus ECF.

The formulas suggested by P.E. Watson (Watson et al., 1980) were used to calculate TBW for each male (E.1) and female (E.2) patient:

(E.1) $$\begin{array}{c} \;{\mathrm{TBW}}_{\text{male}}=2.447–0.09516\times \mathrm{Age}+0.1074\times \text{height}+0.3362\\ \;\times \text{weight}\;\left(\text{liters}\right)\;\left(\mathrm{yr}\right)\;\left(\mathrm{cm}\right)\;\left(\mathrm{kg}\right). \end{array}$$

(E.2) $$\begin{array}{c} {\mathrm{TBW}}_{\text{female}}=-2.097+0.1069\times \text{height}+0.2466\\ \;\times \text{weight}\;\left(\text{liters}\right)\;\left(\mathrm{cm}\right)\;\left(\mathrm{kg}\right) \end{array}$$

Ultrafiltration (UF) was ignored in the calculation of body weight. The individual predialytic body weight was entered into the equations.

The TBW was set to be equal to V_1_ + V_2_ + V_3_ for each male (E.1) or female (E.2) patient based on knowledge about the distribution of physiological molecules in general (Carson & Cobelli, 2014; Costanzo, 2018).

Based on knowledge about fluid distribution in the body, ECF was set to 1/3 of TBW, and plasma was set to 1/4 of ECF (Costanzo, 2018). Based on these assumptions, the distribution volumes of phosphate in the three compartments (V_1_, V_2_, and V_3_) were determined by equations E.3, E.4, and E.5.

(E.3) $$V_{1}=\mathrm{TBW}*1/3*1/4$$

(E.4) $$V_{2}={\mathrm{TBW}}^{*}1/3*3/4$$

(E.5) $$V_{3}=\mathrm{TBW}*2/3$$

#### 2.4.3 Determination of mass transfer coefficients and phosphate clearance

The dialyzer phosphate clearance (k_d_) was set to be equal to the mean dialyzer clearance value for each patient—that is, the value was calculated based on the dialysate phosphate samples from each patient, mean dialysate flow rate, and plasma phosphate concentrations at the time points where dialysate was measured in HD1. (E.6) illustrates the calculation.

(E.6) $$\mathrm{kd}=\frac{\frac{\left(\Sigma \;\text{phosphate conc}.\;\text{in dialysate}\;\right)}{n_{d}}*\text{mean dialysate flow rate}\;}{\frac{\Sigma \;\text{phosphate conc}.\;\text{in plasma}\;}{n_{p}}}$$

The n_p_ and n_d_ components are the number of plasma samples and dialysate samples, respectively. The two mass transfer coefficients (k_1_ and k_2_) were determined for each patient using the *Solver* function in Excel. The *Solver* function was used to obtain the optimum solutions for k_1_ and k_2_ and included minimization of the root mean square error (RMSE) using the measured plasma phosphate concentrations from HD1 and the corresponding modeled plasma phosphate concentrations.

### 2.5 Data analysis and validation

**FIGURE 2:**
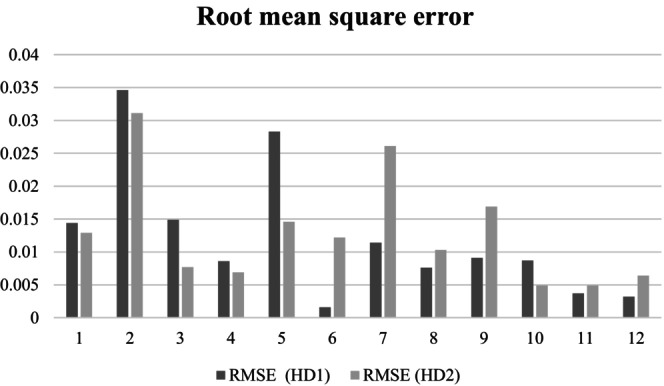
Root mean square error (RMSE) for each of the 12 patients (nos. 1–12) for dialysis 1 (HD1) (dark) and dialysis 2 (HD2) (light).

The goodness of fit to the patient data was calculated for the model simulation showing the lowest RMSE value in each patient for HD1 and HD2, respectively. As described in the previous subsection, the lowest RMSE value was found using the *Solver* function in Excel in each treatment case. The treatment‐specific RMSE results for each patient are illustrated in a double‐bar chart (Figure 2). To assess the goodness of fit to the patient data, the coefficient of determination (R^2^) was determined using the Excel RSQ function that returns the square of the Pearson product–moment correlation coefficient, R, where R is given by equation E.7.

(E.7) $$R=\frac{\sum \left(x-\bar{x}\right)\left(y-\bar{y}\right)}{\sqrt{\sum {\left(x-\bar{x}\right)}^{2}{\left(y-\bar{y}\right)}^{2}}}$$

The inputs *x* and *y* are the two sets of data—that is, measured and modeled plasma phosphate—whereas $\bar{x}$ and $\bar{y}$ are the sample means of the measured and modeled plasma phosphate values, respectively (Excel‐rsq‐function [Internet], 2023).

Using QUARTILE.INC in Excel, median (interquartile range) R^2^ values for HD1 and HD2, respectively, were calculated separately for patients with intradialytic blood samples only (*n* = 8) and separately for those with both intra‐ and postdialytic blood samples (*n* = 4).

Fisher's R to z transformation was used to assess significant difference between HD1 and HD2 model performance (R value) in each patient case. Fisher's R to z transformation produces the observed z value, as given by equation E.8.

(E.8) $$\;z_{\text{observed}}=\frac{\left(z_{1}-z_{2}\right)}{\sqrt{\left[\left(\frac{1}{n_{1}-3}\right)+\left(\frac{1}{n_{2}-3}\right)\right]}}$$

The components z_1_ and z_2_ correspond to the correlation coefficients (R) for HD1 and HD2, respectively—whereas n_1_ and n_2_ correspond to the sample sizes for HD1 and HD2, respectively (Upton & Cook, 2014). A statistically significant difference was concluded when the numerical observed z‐value was higher than 1.96 (significance level, 0.05).

## 3 RESULTS

Table 3 presents the mean values of the measured treatment and patient parameters during HD1 and HD2, respectively. No significant difference was found between the values.

**TABLE 3:** Treatment and patient parameters for the first (HD1) and second (HD2) hemodialysis treatments. The values are expressed as the means±standard deviation calculated from the values of the 12 patients.

Treatment parameter	HD1	HD2
Treatment time (min)	232.5 ± 26.0	230.0 ± 26.6
Blood flow rate (ml/min)	314.2 ± 22.7	303.8 ± 44.3
Dialysate flow rate (ml/min)	445.7 ± 55.4	453.0 ± 72.2
Total fluid removed (l)	1.8 ± 1.0	2.1 ± 1.1
Ultrafiltration rate (ml/min)	7.6 ± 4.0	9.0 ± 4.6
Predialytic plasma phosphate concentration (mmol/l)	1.4 ± 0.4	1.7 ± 0.3
Predialytic body weight (kg)	80.8 ± 14.9	81.3 ± 14.3
Postdialytic body weight (kg)	79.3 ± 15.0	79.6 ± 14.8
Dialyzer phosphate clearance (ml/min)	145.1 ± 24.5	145.0 ± 20.8

Table 4 summarizes the determined parameter values (V_1_, V_2_, V_3_, k_d_, k_1_, and k_2_) for the 12 patients based on the model modification on samples from HD1. The median (interquartile range) values for the coefficients and volumes were (*n* = 12): V_1_ = 3.53 (2.82–3.69) l; V_2_ = 10.57 (8.46–11.07) l; V_3_ = 28.17 (22.56–29.51) l; k_d_ = 8.94 (7.90–9.45) l/h, k_1_ = 17.06 (13.82–31.73) l/h; k_2_ = 12.43 (7.15–23.05) l/h.

**TABLE 4:** Determined patient values for volumes of distribution in the three compartments (V_1_, V_2_, and V_3_), dialyzer clearence (k_d_), and mass transfer coefficients (k_1_ and k_2_). Patients marked with * had undergone both intra‐ and two‐hours postdialytic sampling.

Patient no.	V_1_(l)	V_2_(l)	V_3_(l)	k_d_(l/h)	k_1_(l/h)	k_2_ (l/h)
1	2.82	8.46	22.57	10.12	15.89	26.64
2*	2.72	8.16	21.75	6.77	11.91	1.78
3*	3.02	9.07	24.19	8.88	7.36	782.50
4	3.62	10.85	28.93	9.33	38.50	17.08
5*	3.45	10.34	27.57	9.80	11.07	894.15
6	2.81	8.44	22.51	9.23	21.50	9.23
7	4.36	13.07	34.86	6.63	17.56	21.85
8*	3.60	10.79	28.77	8.99	16.55	10.01
9	3.87	11.62	30.99	11.26	70.34	14.84
10	3.69	11.07	29.51	8.27	29.47	7.30
11	2.45	7.35	19.61	6.48	14.46	6.15
12	3.69	11.07	29.51	8.72	53.15	6.68

Table 5 summarizes, for each patient, the *R* ^2^ and RMSE values for HD1 and HD2. Considering patients with intradialysis values only, the median (interquartile range) *R* ^2^ values were 0.985 (0.959–0.997) for HD1 and 0.992 (0.984–0.994) for HD2. Considering patients with both intra‐ and postdialysis values, the median (interquartile range) *R* ^2^ values were 0.882 (0.838–0.929) for HD1 and 0.963 (0.951–0.976) for HD2. Eight of the 12 patients, three of whom with both intra‐ and postdialytic values, demonstrated higher *R* ^2^ values for HD2 than for HD1 when comparing the simulations with the data. A statistically significantly higher (|z obs. value| > 1.96) *R* ^2^ value for HD2 was found for one patient. A graphical illustration of the RMSE values is provided in Figure 2 where it can be seen that for six of the 12 patients lower RMSE values for HD2 than for HD1 was found.

**TABLE 5:** Treatment time, coefficient of determination (*R* ^2^) and root‐mean‐square error (RMSE) for hemodialysis 1 (HD1) and hemodialysis 2 (HD2) in each patient case (*n* = 12) together with the observed z value (z obs.) for each patient.

		HD1		HD2			*Z obs*
Patient no.	Treatment time HD1 (min)	RMSE	R^2^ (95% CI)	Treatment time HD2 (min)	RMSE	R^2^ (95% CI)	
1	240	0.0144	0.906	240	0.0129	0.984	−1.58
2*	240	0.0346	0.852	210	0.0311	0.990	−2.98**
3*	240	0.0149	0.912	240	0.0077	0.955	−0.77
4	240	0.0086	0.968	240	0.0069	0.983	−0.54
5*	270	0.0283	0.794	270	0.0146	0.939	−1.53
6	240	0.0016	0.999	240	0.0122	0.992	1.94
7	240	0.0114	0.931	240	0.0261	0.969	−0.71
8*	240	0.0076	0.981	240	0.0103	0.971	0.48
9	180	0.0091	0.987	180	0.0169	0.993	−0.45
10	240	0.0087	0.982	240	0.0049	0.995	−1.08
11	240	0.0037	0.997	240	0.0049	0.995	0.51
12	240	0.0032	0.997	240	0.0064	0.991	0.87

*Note*: Patients marked with * had undergone both intra‐ and two‐hours postdialytic sampling, whereas patients without a mark had only undergone intradialysis. Statistically significant differences between R^2^ values are indicated by an observed z value numerically higher than 1.96 and marked with **.

**FIGURE 3:**
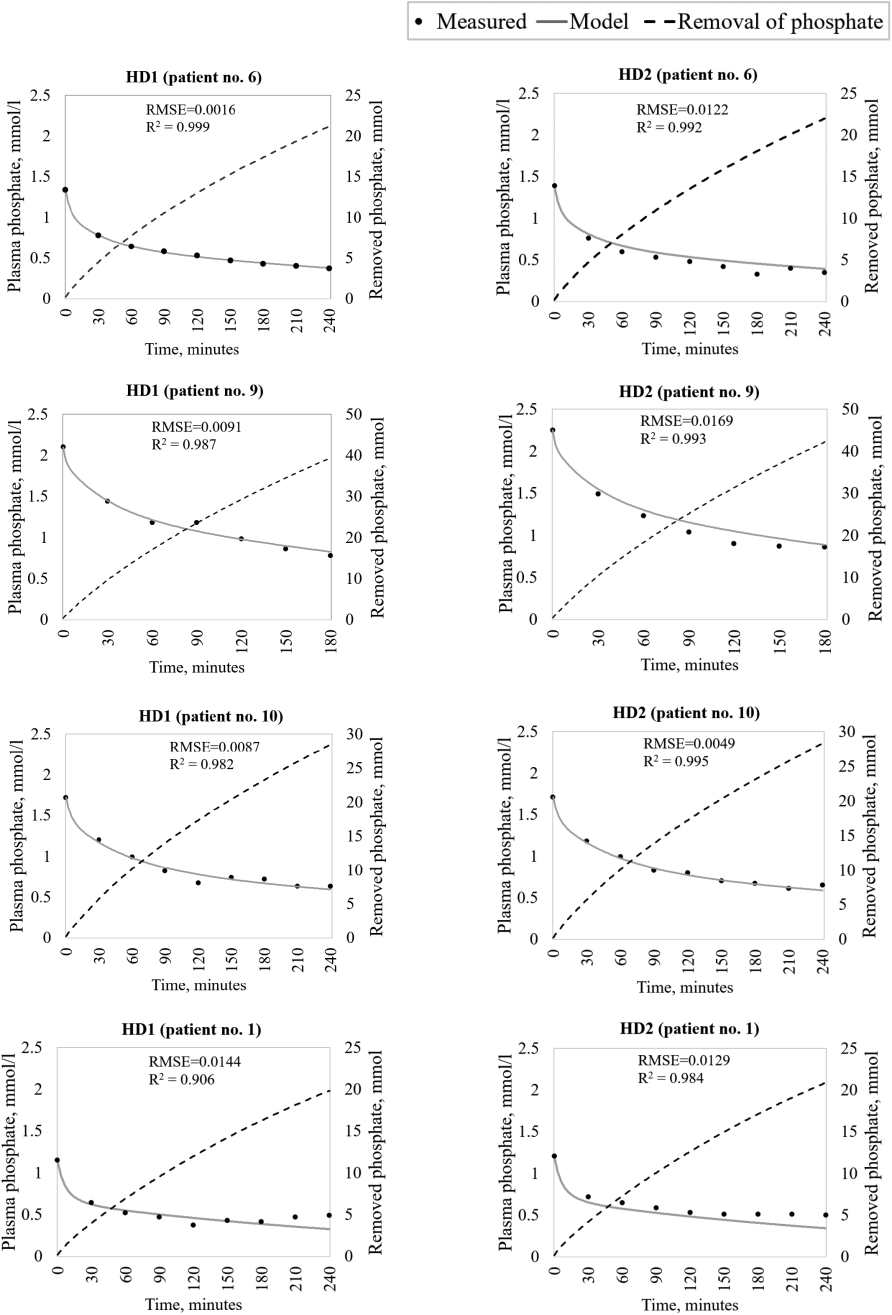
Modeled and measured plasma phosphate concentrations (mmol/l) (left axis) and removed phosphate (mmol) (right axis) for hemodialysis 1 (HD1; left) and 2 (HD2; right) for four selected patients with intradialytic measurements only. (a) Illustrates the simulations for the patient (no. 6) with the highest mean coefficient of determination (R^2^) value based on HD1 simulations. (b, c) illustrate the patients with the fourth (patient no. 9) and fifth (patient no. 10) best R^2^ values, respectively, based on HD1 simulations. (d) Is the patient with the lowest goodness of fit in HD1.

**FIGURE 4:**
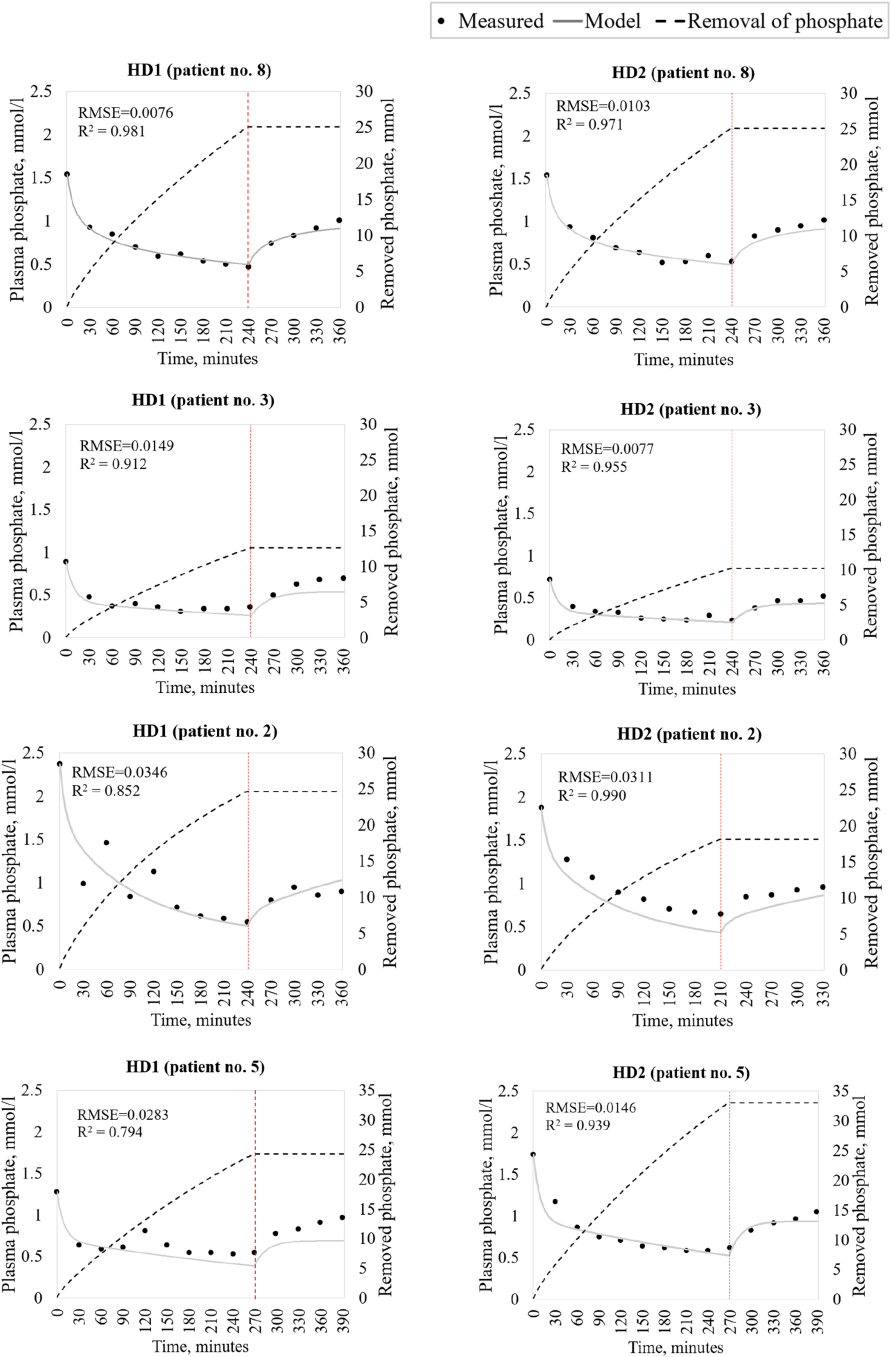
Modeled and measured plasma phosphate concentrations and removed phosphate for hemodialysis 1 (HD1; left) and 2 (HD2; right) for three selected patients with both intra‐ and postdialytic measurements. (a) Illustrates the simulations for the patient (no. the simulations for the patient (no. 8) with the highest mean coefficient of determination (R^2^) value based on HD1 simulations. (b, c) illustrate the patients with the second (patient no. 3) and third (patient no. 2) best R^2^ values, respectively, based on HD1 simulations. (d) Is the patient with the lowest goodness of fit in HD1. The dotted red vertical line illustrates the termination of dialysis.

Figure 3 provides examples from four patients with intradialytic measurements only (i.e., 4 of 8 patients), illustrating the graphical agreement for HD1 and HD2 between the measured plasma phosphate and model simulations. The figure also provides information about phosphate removal in each treatment. Graphs are shown from the patient demonstrating the highest goodness of fit in HD1 (patient no. 6; *R* ^2^ = 0.999), two patients with a typical goodness of fit in HD 1 (patient no. 9: *R* ^2^ = 0.987; patient no. 10: *R* ^2^ = 0.982; the two patients with *R* ^2^ closest to the median *R* ^2^ for patients with intradialytic measures only), and the patient demonstrating the lowest goodness of fit in HD1 (patient no. 1: *R* ^2^ = 0.906). Figure 4 provides similar examples from four patients (i.e., all patients) with both intra‐ and postdialytic plasma phosphate measurements, illustrating the graphical agreement for HD1 and HD2. The graphical results for all HD1 and HD2 treatments (*n* = 24) are available in Data S2.

Table 6 summarizes, for each patient, the modeled and measured phosphate removal for HD1 and HD2. Mean ± SD modeled phosphate removal was 24.38 ± 6.20 mmol (95% CI: 20.87–27.89) and 25.03 ± 8.00 mmol (95% CI: 20.50–29.56) for HD1 and HD2, respectively. Mean ± SD measured phosphate removal was 22.25 ± 5.21 mmol (95% CI: 19.302–25.198) and 21.55 ± 5.90 mmol (95% CI: 18.212–24.888) for HD1 and HD2, respectively. No significant differences were found between modeled phosphate removal between treatments (i.e. HD1 and HD2) or between modeled and measured phosphate removal.

**TABLE 6:** Dialysis duration (min) and modeled and measured phosphate removal (in mmol) for hemodialysis 1 (HD1) and 2 (HD2) for each of the 12 patients.

Patient no.	Dialysis duration (min)HD1/HD2	HD1		HD2	
		Modeled removal (mmol)	Measured removal (mmol)	Modeled removal (mmol)	Measured removal (mmol)
1	240/240	19.84	18.12	20.88	20.64
2	240/210	24.69	25.44	18.17	16.74
3	240/240	12.67	12.70	10.25	10.04
4	240/240	26.49	23.42	28.86	20.78
5	270/270	24.33	26.73	33.07	24.81
6	240/240	21.18	19.00	21.97	23.76
7	240/240	23.55	22.16	25.13	14.84
8	240/240	25.11	22.03	25.11	28.17
9	180/180	39.38	33.09	42.20	26.55
10	240/240	28.44	24.90	28.27	18.54
11	240/240	21.83	17.36	20.07	22.89
12	240/240	25.06	22.08	26.43	30.8

## 4 DISCUSSION

This study aimed to modify and validate a promising three‐compartment phosphate kinetic model (Laursen et al., 2015) in HD therapy based on individual patient data (*n* = 12, two treatments from each patient, HD1 and HD2) and to assess the temporal robustness of model predictions. The material included data from 12 patients: The data from eight of the patients included intradialytic samples only. The remaining four data sets included both intradialytic and 2‐h postdialytic samples. The method consisted of two steps: First, the model was modified and validated on HD1 samples and included individualization of the six components V_1_, V_2_, V_3_, k_d_, k_1_, and k_2_. Second, the model was validated on HD2 samples without fitting or changing the components.

Based on the median (interquartile range) *R* ^2^ values of 0.985 (0.959–0.997) for HD1 and 0.992 (0.984–0.994) for HD2 found in the study, the three‐compartment model seems promising in simulating intradialytic phosphate kinetics in individual HD patients, supporting the results based on mean plasma phosphate samples in our previous paper (Laursen et al., 2015). A high goodness of fit is concluded as the *R* ^2^ values are similar or higher when compared to results from other modeling studies (Agar et al., 2011; Maasrani et al., 1995). For instance, Maasrani et al. reported good agreement based on R^2^ values between 0.813 and 0.992 when considering intradialytic phosphate kinetics (Maasrani et al., 1995). In our study, the best agreement was found when fitting the model to HD1 data sets with only intradialytic plasma phosphate concentrations. When fitting the model to HD1 data sets with both intra‐ and postdialytic samples, a somewhat lower agreement was found with a median (interquartile range) *R* ^*2*^ value of 0.882 (0.838–0.929). In particular, the disagreement for HD1 was evident in the second hour after the termination of dialysis for patients 3 and 5 (Figure 4). By contrast, the fit to HD2 data for those two patients, with the model fitted to HD1, was visibly better. Although only based on data from four patients, and although it might be due to unexplained variations in data, these postdialytic discrepancies in HD1 could indicate the need for further modifications of the model. One approach could be to incorporate some of the model components presented by some of the authors in a previous systematic review (Laursen et al., 2017). Examples include UF, residual renal clearance, and hematocrit values. UF would be relevant to account for the convective flux of phosphate (Andersen & Bangsgaard, 2023; Spalding et al., 2002), whereas residual renal function could play a significant role considering phosphate elimination in patients who still excrete phosphate through the kidneys. For instance, in a study by Iwasawa et al. it was found that the amount of phosphate removed by the residual kidney was approximately 1.5‐fold greater per week in patients with a glomerular filtration rate >3 mL/min compared to the phosphate removal during one HD treatment (Iwasawa et al., 2013). Regarding hematocrit values, different studies (Lim et al., 1990; Ronco et al., 2001) have found that phosphate removal is significantly reduced by increased hematocrit levels during HD. This is especially prominent in patients undergoing UF since hemoconcentration resulting from UF causes a progressive increase in hematocrit during dialysis (Clark et al., 2007). Thus, these components could have influenced our modeling results and should be considered in the further development of the model. Moreover, variations in dialyzer clearance may play a role. In addition to the dialyzer type, we could consider the influence of coagulation on dialyzer fibers and membrane pores. Independent studies have indicated that dialyzer clearance is interconnected with intradialytic blood clotting (Kuhlmann, 2010; Suranyi & Chow, 2010). Intradialytic blood clotting could also be a factor in the variation in the intrapersonal patient data. Other factors that affect the variation may be related to the dialysate and blood flow rates, dialysis access type, and unregistered meals—factors that were not included in the model. Another approach to optimize the model approach could be to add one or more compartments to the model, as suggested by Spalding et al. (Spalding et al., 2002). Additionally, the model may have encountered a critical phosphate limit, triggering a rise in the plasma phosphate concentration from an undefined compartment. This phenomenon would be consistent with former speculations (Pogglitsch et al., 1989; Spalding et al., 2002; Sugisaki et al., 1983). Modeling of a fourth compartment could be based on nuclear magnetic resonance studies demonstrating that glycophosphates in erythrocytes may contribute to phosphate during dialysis (Pogglitsch et al., 1989; Sugisaki et al., 1983). It might be noted that the modeled (and measured) phosphate removal values are consistent with results from different studies investigating conventional HD phosphate removal (Wang et al., 2012; Zhang et al., 2021).

According to the above, it could have been relevant to consider other parameters to ensure a more accurate and reliable model. However, as stated in the previous systematic review by the authors (Laursen et al., 2017), high accessibility is essential if models are to succeed in practical settings, which would command simplicity and transparency. To achieve simplicity, the model should not evaluate on too many parameters. Therefore, it must always be assessed whether one must compromise with accuracy or accessibility in the development of the model.

Good temporal robustness is important, for example, when the model is fitted to data from one HD treatment and then used to make clinical decisions regarding subsequent HD treatments for a given patient. Our results, in general, did not show lower *R* ^2^ values and higher RMSE values for HD2 than those values for HD1 and no significant difference was found between the HD1 and HD2 values. These results indicate good temporal robustness of the model predictions. However, the result could also be explained by similarity in phosphate behavior between the weeks rather than by robustness of the model. In this relation, it is considered that non‐significant variations in the data could explain that higher *R* ^2^ values for eight of the 12 HD2 simulations were obtained than those for HD1. Also, it should be noted that for half of the simulations, lower RMSE values for HD2 than for HD1 was found. Therefore, because any model, without fitting the model to new data, will likely have a decay in performance over time, it may be suggested that 1 week may be too short to observe such changes when analyzing data from the relatively small number of patients included in our study. Hence, it would be relevant to perform further studies with a longer duration between the two treatments. It could also be relevant to consider fitting the model based on the first 2–3 time points to predict phosphate kinetics and removal for the remaining HD time. A well‐working model with good predictive abilities could thus provide the clinicians with a valuable tool for instance considering if the individual patient could benefit from prolonged treatment.

Our results showed that only one in three patients agreed to participate in postdialysis data collection. This is a significant limitation of the present study, since phosphate is assumed to be generated and released from additional compartments, when levels reach critically low levels in the later stages of HD leading to a rebound of phosphate in the postdialytic phase (Laursen et al., 2017; Spalding et al., 2002). Thus, postdialytic data, should have more focus in future studies. Additionally, it would be very relevant to validate the model on samples from other treatment regimens. For example, it could be interesting to test the model on prolonged dialysis. In the present study, the length of HD was between 180 and 270 min—that is, longer dialysis such as nocturnal HD was not considered. In our previous model study (Laursen et al., 2015), the model was fitted to eight‐hour HD with promising results. However, the validation was limited to mean patient values. Hence, it would be highly relevant to verify the results in a future study on individual patient data in prolonged dialysis. Furthermore, it could also be interesting to test the robustness of the model on hemodiafiltration (HDF) treatment data. This perspective is relevant because it is well‐known that HDF treatment produces higher phosphate removal during treatment than HD therapy (Kuhlmann, 2006).

Regarding the model components and coefficients, V_1_, V_2_, V_3_, k_d_, k_1_ and k_2_, the model is partly empirical and partly theoretical. Hence, the model follows physiological expectations when considering the calculation of the volumes of distribution (V_1_, V_2_, and V_3_) and the dialysis clearance (k_d_). However, a few of the mass transfer coefficients (k_1_ and k_2_) values were rather extreme and thus unlikely. The k_2_ parameters for patients 3 and 5 seem to be physiologically unlikely compared with those for the other 10 patients. Patients 3 and 5 were two of the four patients with both intra‐ and postdialytic samples, likely indicating that, for this type of data, further modifications of the model are needed.

In conclusion, the three‐compartment model seems promising in simulating individual intradialytic phosphate kinetics and showed good temporal robustness of model predictions. However, further studies should include a longer follow‐up period between treatments to verify the results. Evenso, the model seems to have potential to provide a quantitative tool to support clinical decisions regarding dialysis phosphate removal; for instance considering if the individual patient could benefit from prolonged treatment. If the model is used to analyze postdialytic phosphate kinetics, particular emphasis should be placed on potential modifications that may be needed for this condition. Further studies should involve more intradialytic and postdialytic data.

## AUTHOR CONTRIBUTIONS

The data collection was carried out by Sisse H. Laursen and Lise Boel with support from Lisbet Brandi, and Jeppe H. Christensen. Sisse H. Laursen wrote the manuscript and did the analysis with support from Ole Kristian Hejlesen. All authors contributed to the final manuscript and provided critical feedback during the process. The study was supervised by Ole Kristian Hejlesen and Peter Vestergaard.

## FUNDING INFORMATION

This work was supported by research grants from The Danish Diabetes Academy supported by the Novo Nordisk Foundation. Grant number OL8201.

## CONFLICT OF INTEREST STATEMENT

The authors declare no conflicts of interest.

## Ethics Statement

All procedures in this study were conducted in accordance with the Danish National Ethics Committee’s approved protocols. Verbal and written informed consent was obtained from all participants for their anonymized data and information to be published in this article.

## Supporting information


Data S1.

## Data Availability

Data not available—participant consent.
