# Supplementary material for: Evaluation of a phosphate kinetics model in hemodialysis therapy—Assessment of the temporal robustness of model predictions
Source: Physiol Rep. 2023 Dec 21;11(24):e15899. doi: 10.14814/phy2.15899 (PMC10737683; doi:10.14814/phy2.15899)

**Supplementary material 1**

**S1.** Model components.

| Model component | Description | Unit | Components and/or equations |
| --- | --- | --- | --- |
| C_1_ | Phosphate concentration in compartment 1  (plasma conc.) | mmol/l | ${\text{ C}_{\text{1}}\text{(t+1)}\text{ }}_{\text{ }}\text{= }$ $\frac{\text{M}_{\text{1}}\text{(t)-( f1(t)+ f2(t))× ((t+1)-(t))}}{\text{V}_{\text{1}}}$ |
| C_2_ | Phosphate concentration in compartment 2 | mmol/l | Two-compartment model:  $\text{C}_{\text{2}}\text{(t+1)\_a}$ $\text{= }\frac{\text{M}_{\text{2}}\text{(t)+ f2(t) }\text{×((t+1)-(t)}\text{)}}{V_{\text{2}}}$  Three-compartment model:  ${\text{C}_{\text{2}}\text{(t+1)\_b}}_{\text{ }}= \frac{M_{2}\left( t \right)+\left( f2\left( t \right)-f3\left( t \right) \right) \times\text{((t+1)-(t))}}{V_{2}}$ |
| C_3_ | Phosphate concentration in compartment 3 | mmol/l | $\text{C}_{\text{3}}\text{(t+1)}\text{ =} \frac{\text{M}_{\text{3}}\text{(t)+ f2(t) ×}\text{ ((t+1)-(t)}\text{)}}{\text{V}_{\text{3}}}$ |
| M_1_ | Mass of phosphate in compartment 1 | mmol | M_1_(t+1) = $\text{M}_{\text{1}}\text{(t)-}\text{( f1}\text{(t)+ f2(t)) × ((t+1)-(t))}$ |
| M_2_ | Mass of phosphate in compartment 2 | mmol | Two-compartment model:  M_2_(t+1)$\text{= M}_{\text{2}}\text{(t)+ f2(t) × ((t+1)-(t))}$  Three-compartment model:  ${M_{2}(t+1)= M}_{2}\left( t \right)+\left( f2\left( t \right)-f3\left( t \right) \right)\times\text{((t+1)-(t))}$ |
| M_3_ | Mass of phosphate in compartment 3 | mmol | ${M_{3}(t+1)\text{= M}}_{\text{3}}\text{(t)+ f2(t) × ((t+1)-(t))}$ |
| TBW | Total body water | l | TBWmale = 2.447 − 0.09516 Age + 0.1074 height + 0.3362 weight  TBWfemale = −2.097 + 0.1069 height + 0.2466 weight |
| V_1_ | Volume of distribution in compartment 1 | l | V_1_ = TBW * 1/3 * 1/4 |
| V_2_ | Volume of distribution in compartment 2 | l | V_2_= TBW *1/3 * 3/4 |
| V_3_ | Volume of distribution in compartment 3 | l | V_3_ = TBW * 2/3 |
| *f_1_* | Phosphate eliminated through dialysis clearance | mmol/  min | f_1_(t) = k_d_ $\text{×}$ (C_1_(t)-C_d_(t)) $\text{×}$ s |
| *f_2_* | Phosphate diffused between compartment 1 and 2 | mmol  /min | f_2_(t) = k_1_ $\text{×}$ (C_1_(t)-C_2_(t)) |
| *f_3_* | Phosphate diffused between compartment 2 and 3 | mmol/  min | f_3_(t)= k_2_ $\text{×}$ (C_2_(t)-C_3_(t)) |
| n_d_ | Number of individual dialysate samples | - | Observed |
| n_p_ | Number of individual plasma samples | - | Observed |
| k_d_ | Dialyzer clearance  (Patient specific) | l/h | k_d_ = $\frac{\left( \Sigma phosphate conc. in dialysate \right) * mean dialysate flow rate}{\Sigma phosphate conc. in plasma}$ |
| k_1_ | Mass transfer coefficient 1  (Patient specific) | l/h | Estimated using the Excel Solver function |
| k_2_ | Mass transfer coefficient 2  (Patient specific) | l/h | Estimated using the Excel Solver function |
|  | Dialysis status | none | (0 = no, 1= yes) |

**Supplementary material 2**

**S2**. The graphical results for hemodialysis 1 (HD1) (left) and hemodialysis 2 (HD2)(right) in each patient case (n=12). The dotted red vertical line in some of the graphical illustrations indicates termination of dialysis.


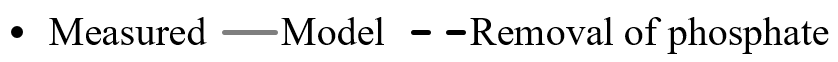


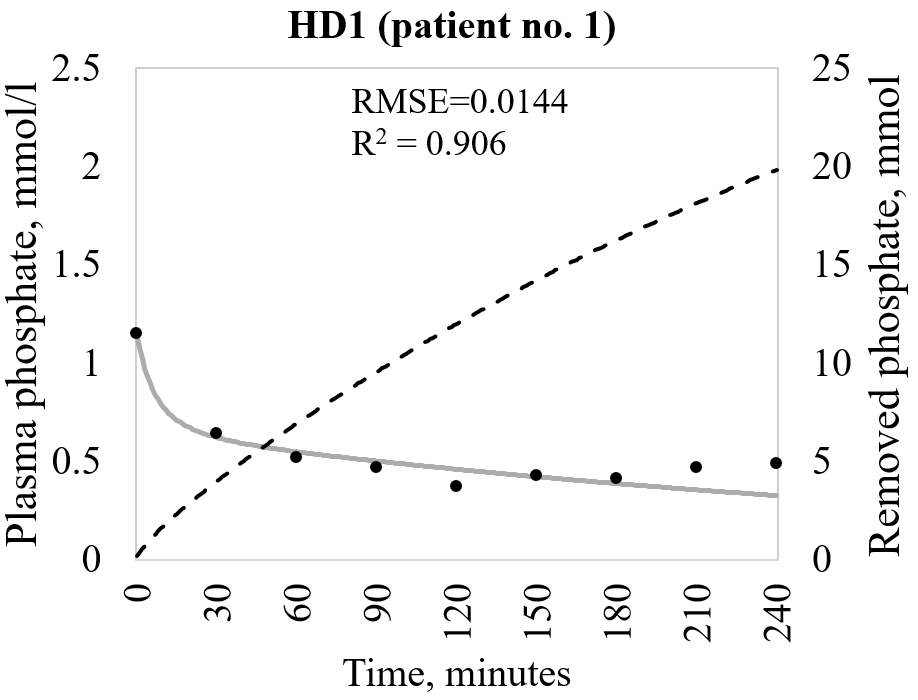

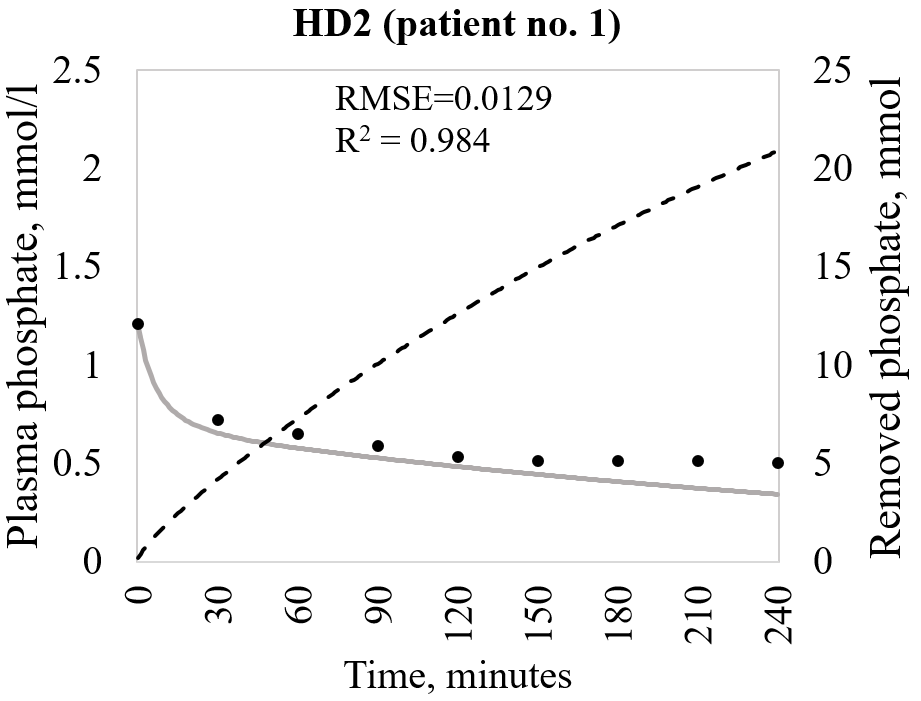


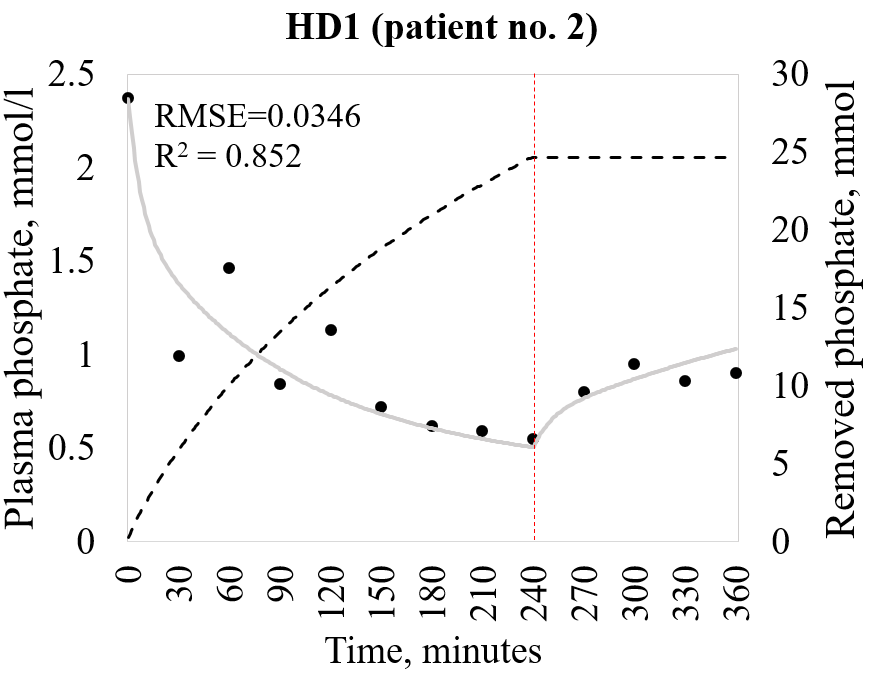

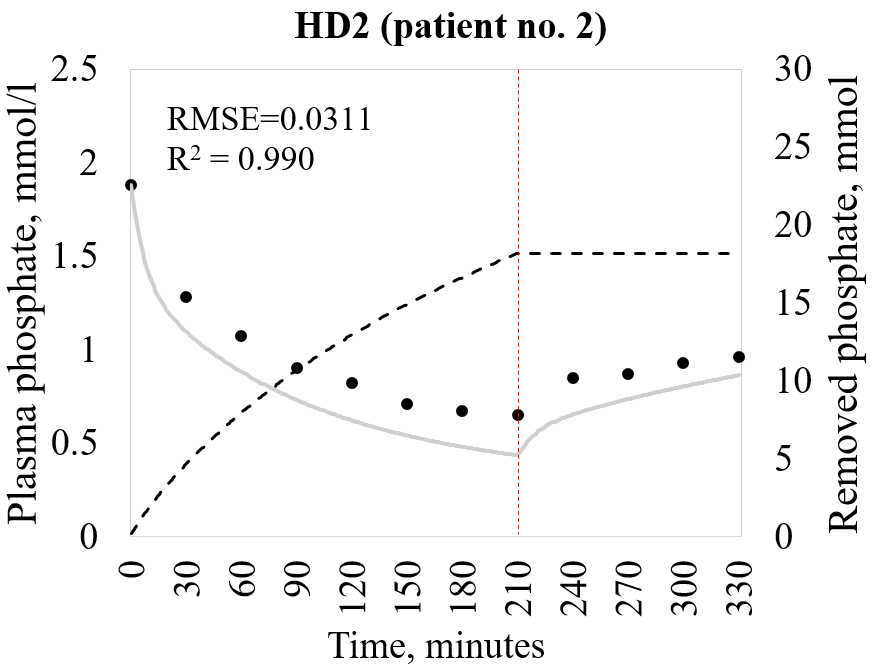


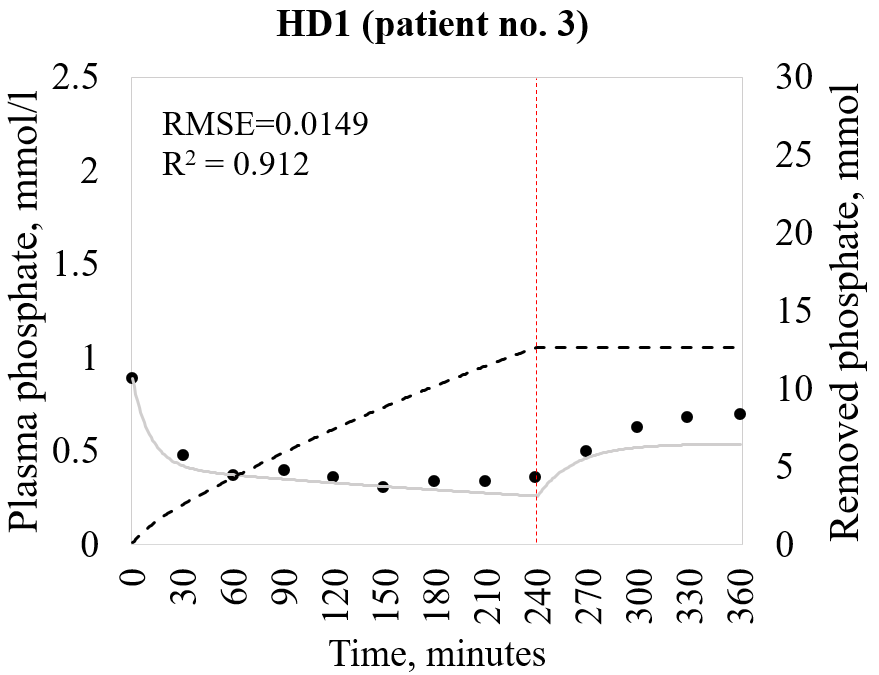

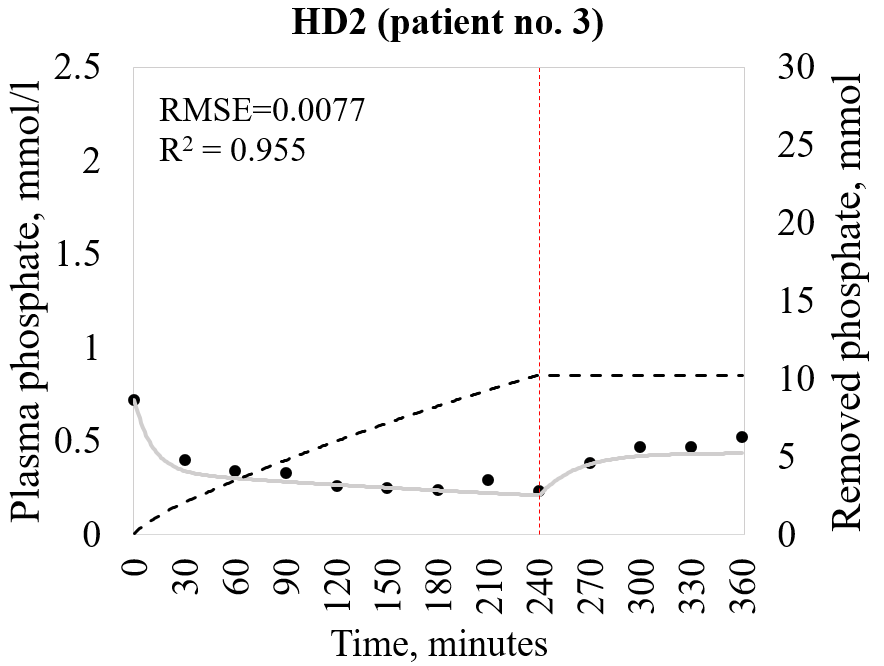


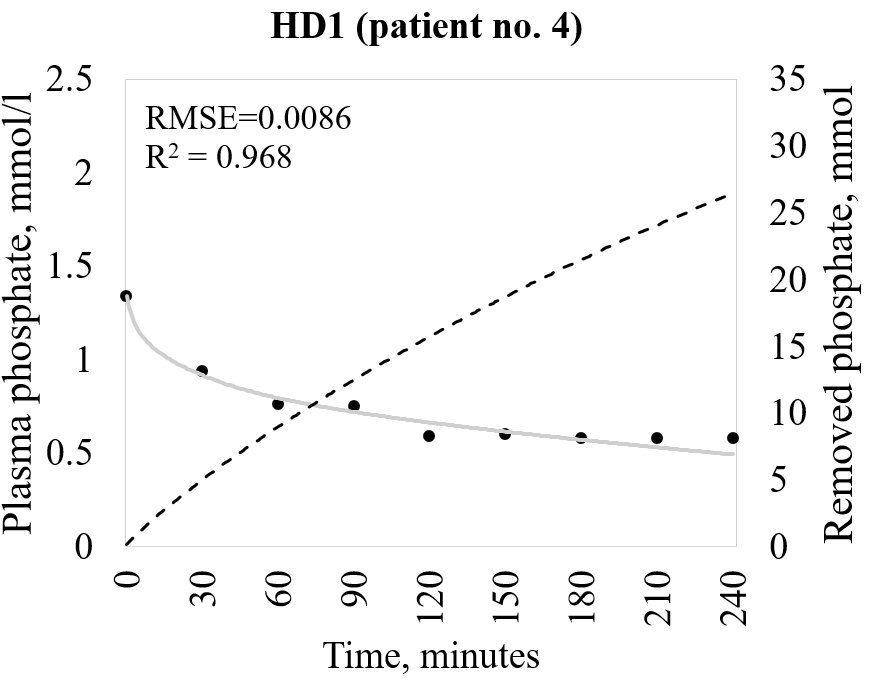

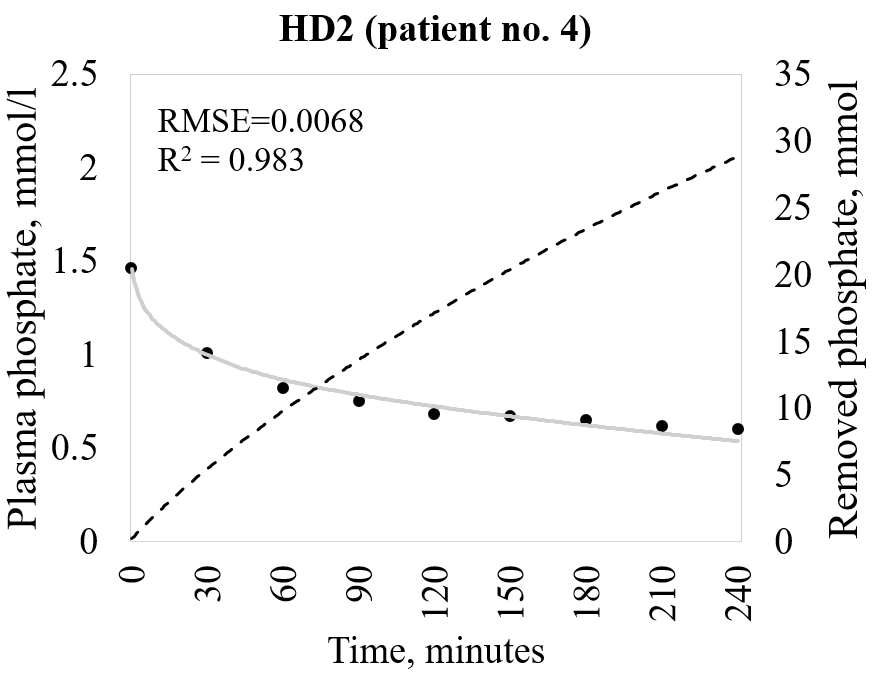


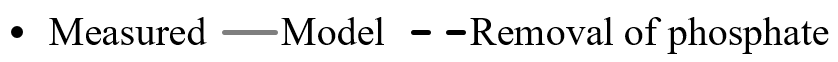


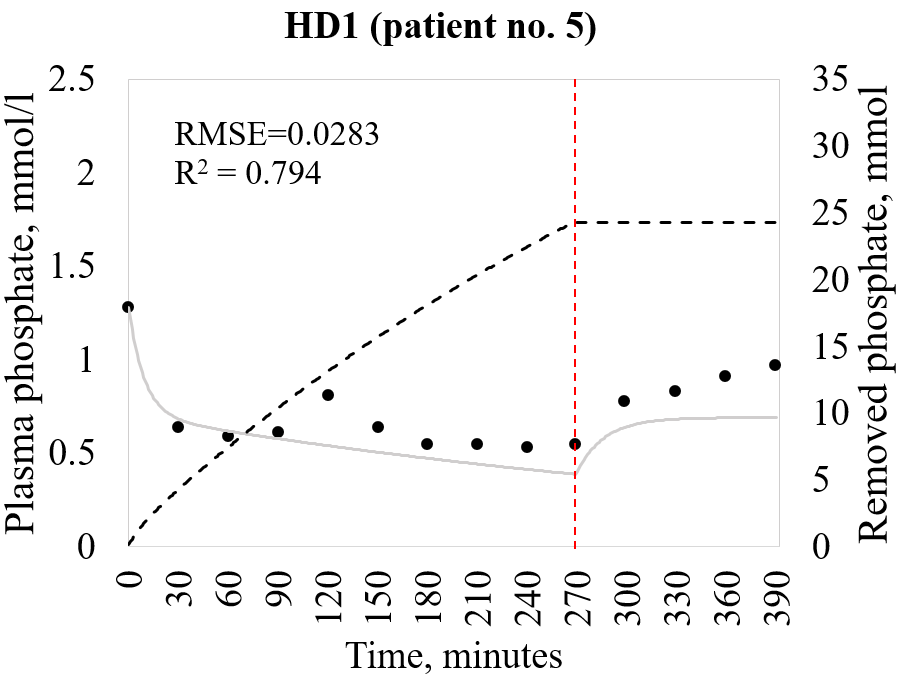

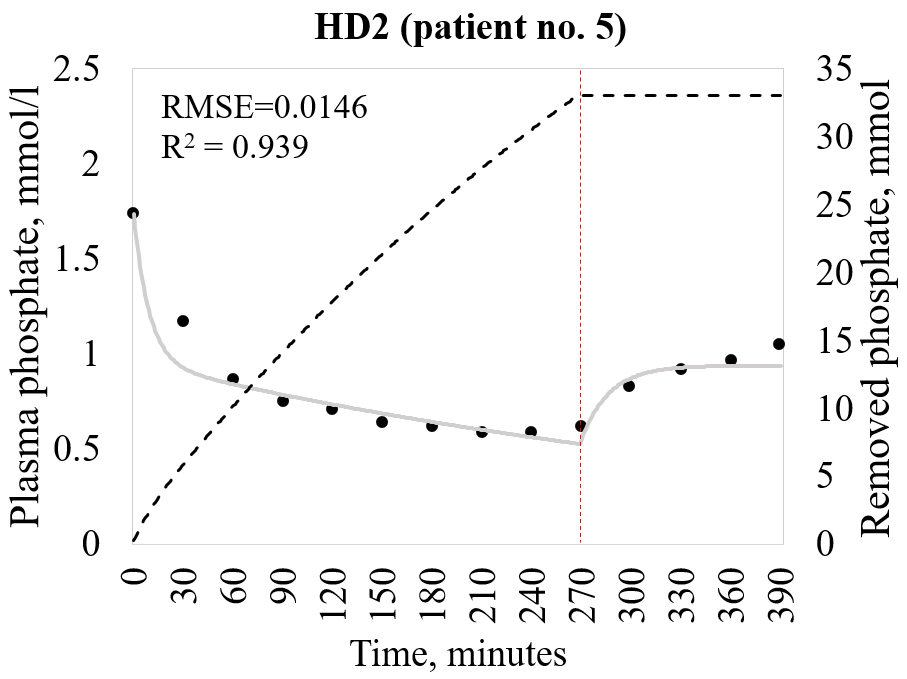


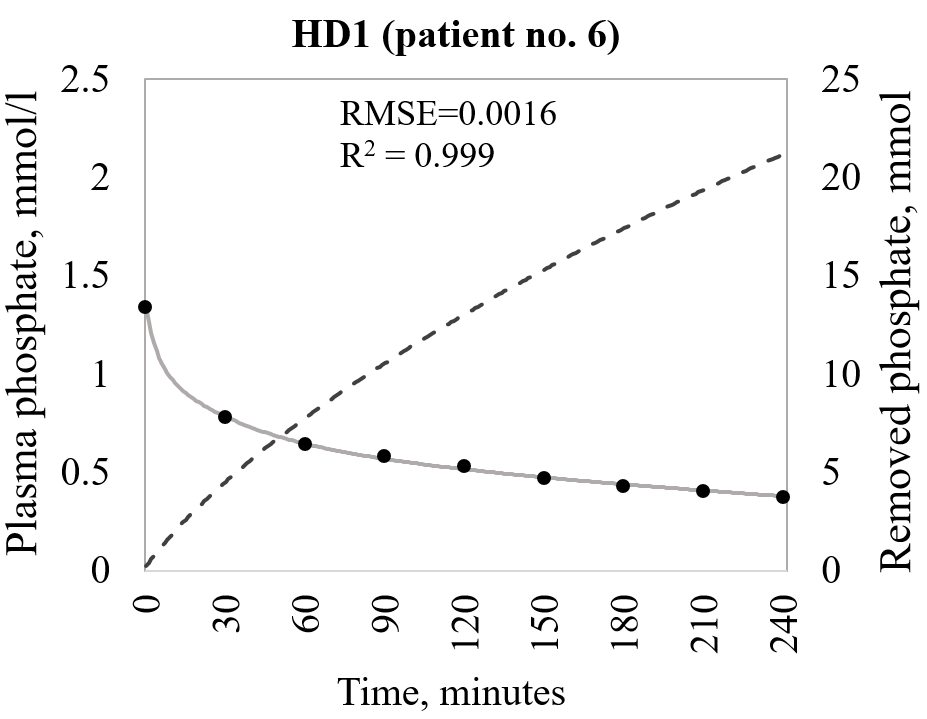

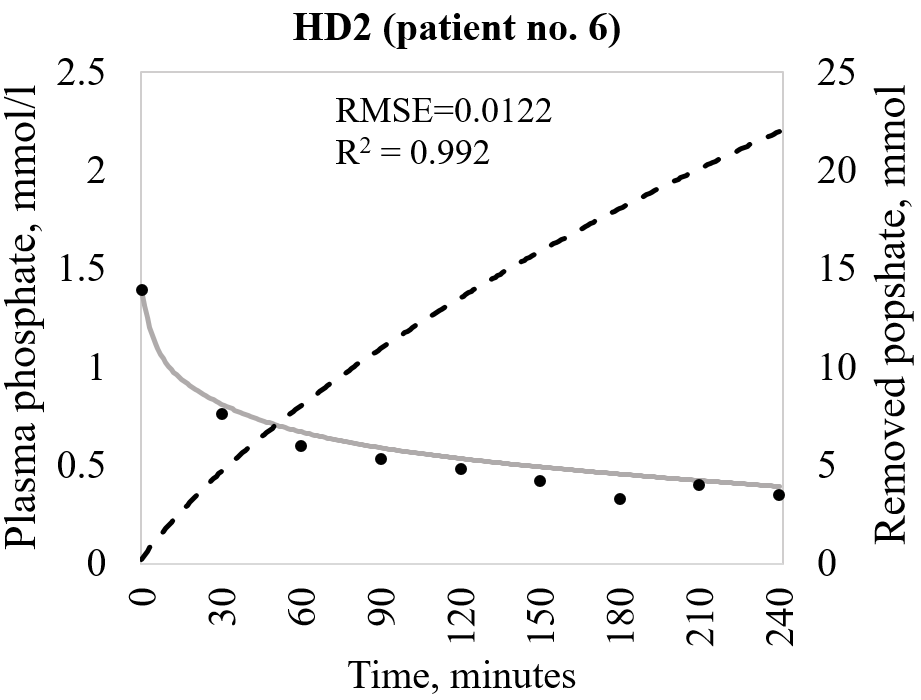


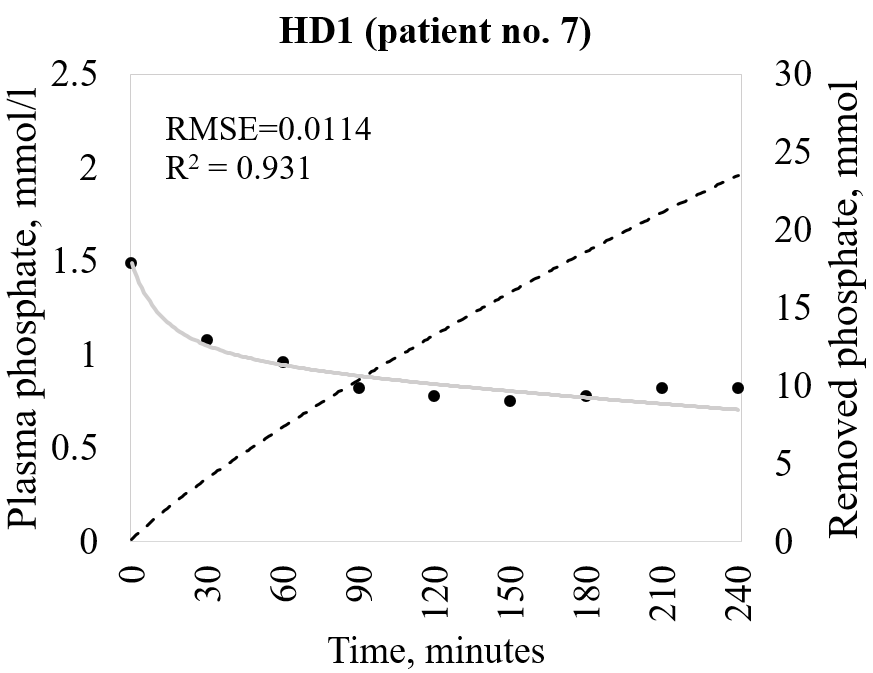

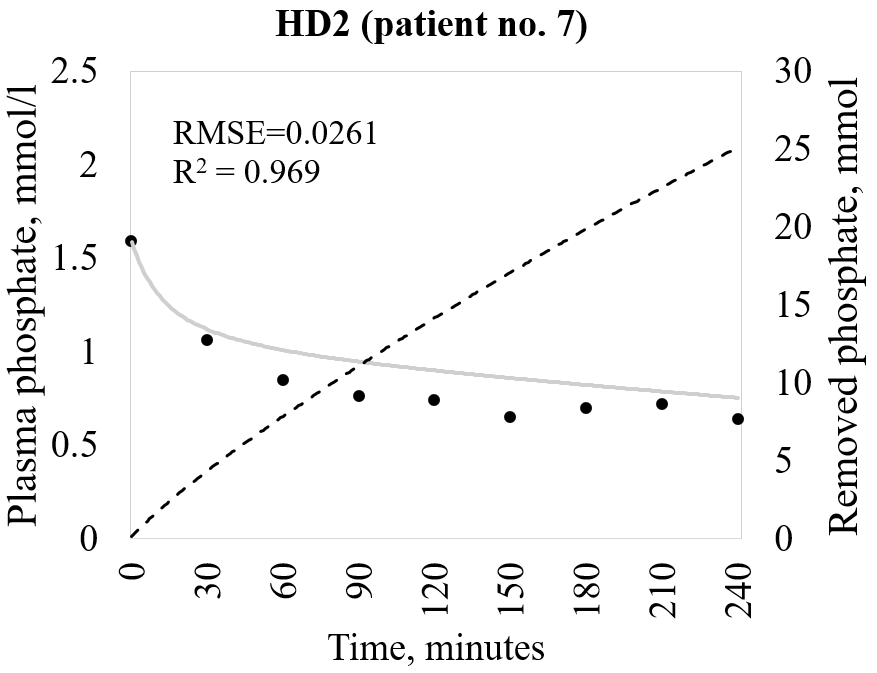


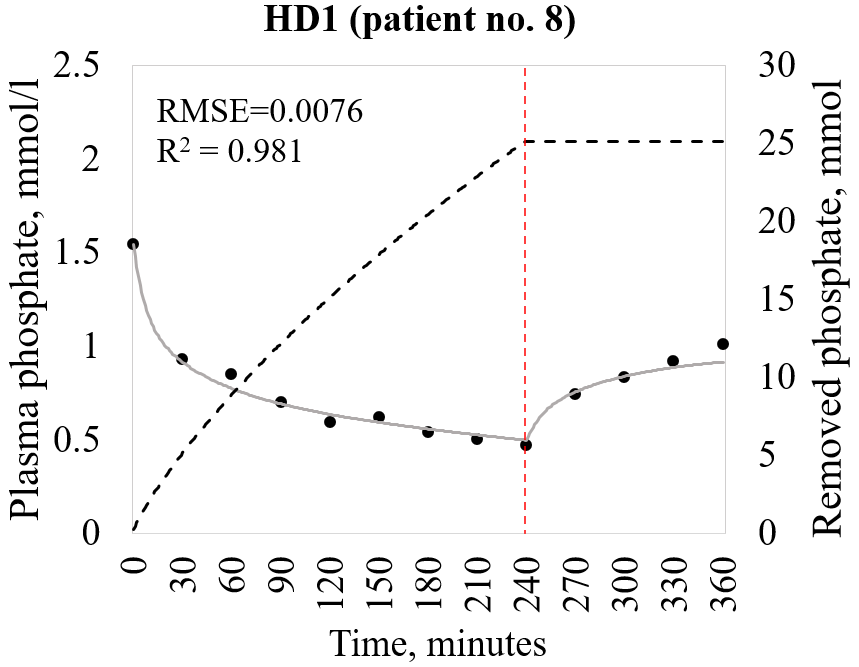

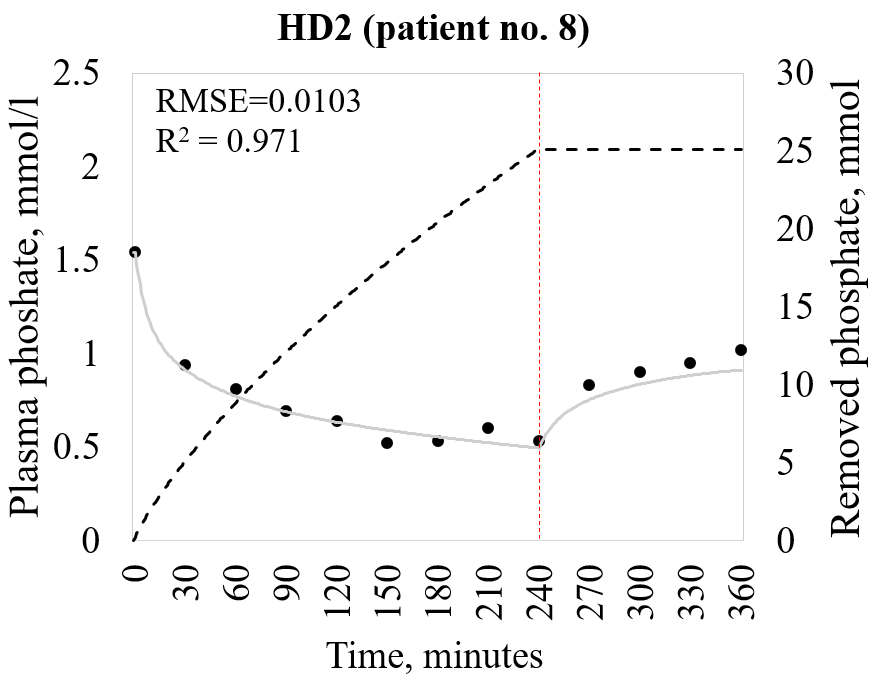


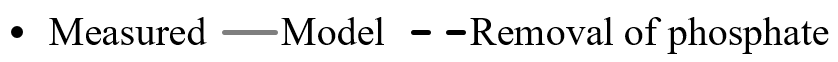


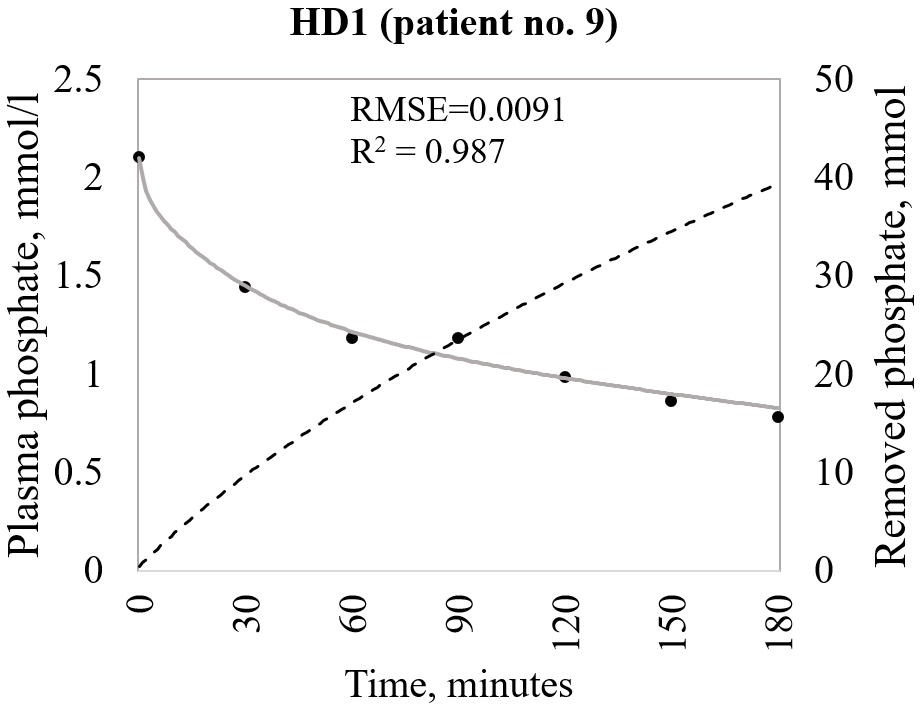

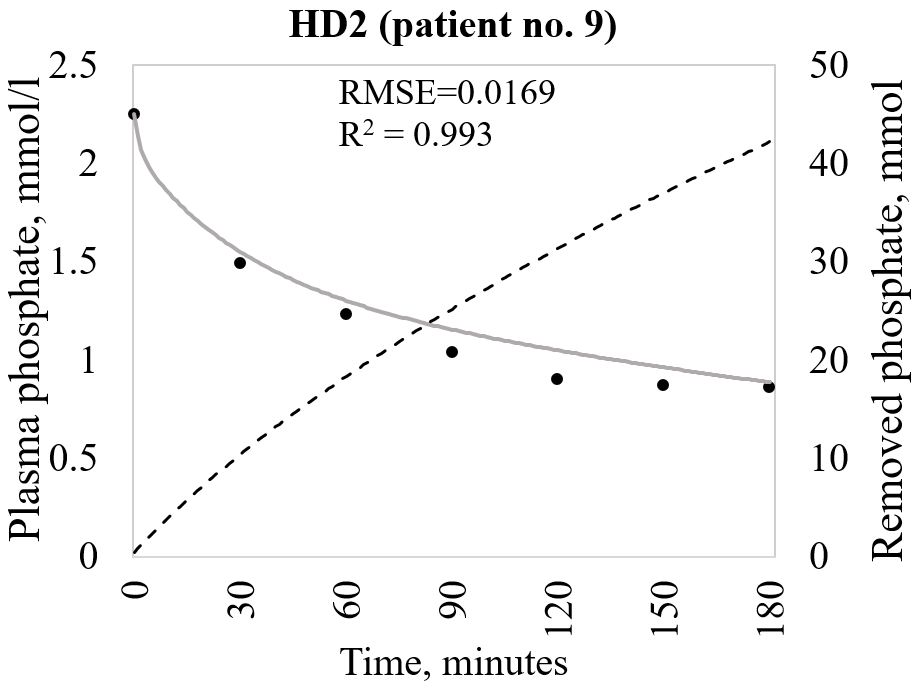


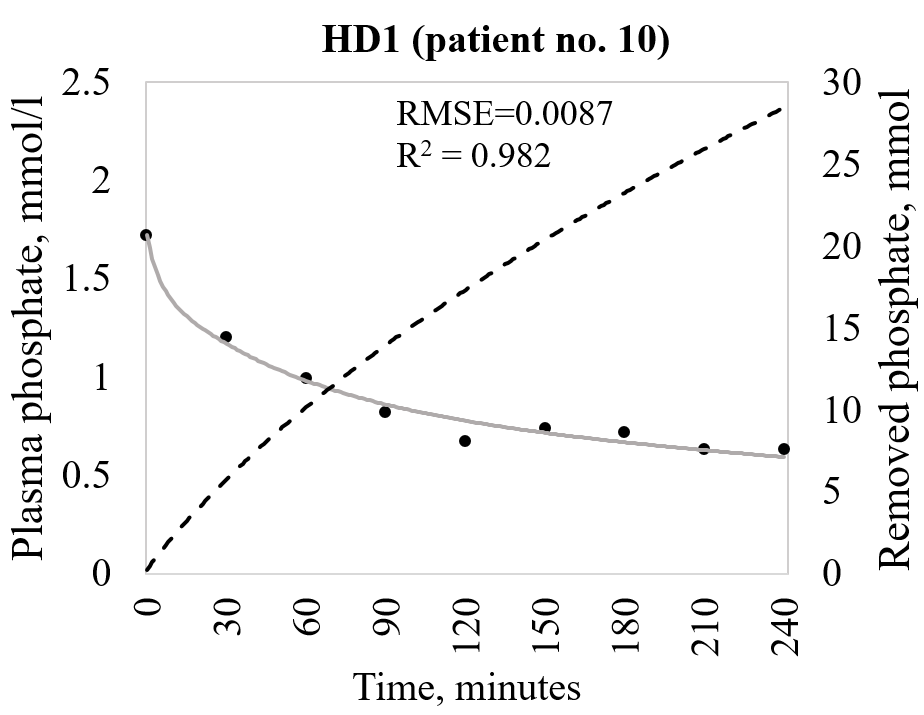

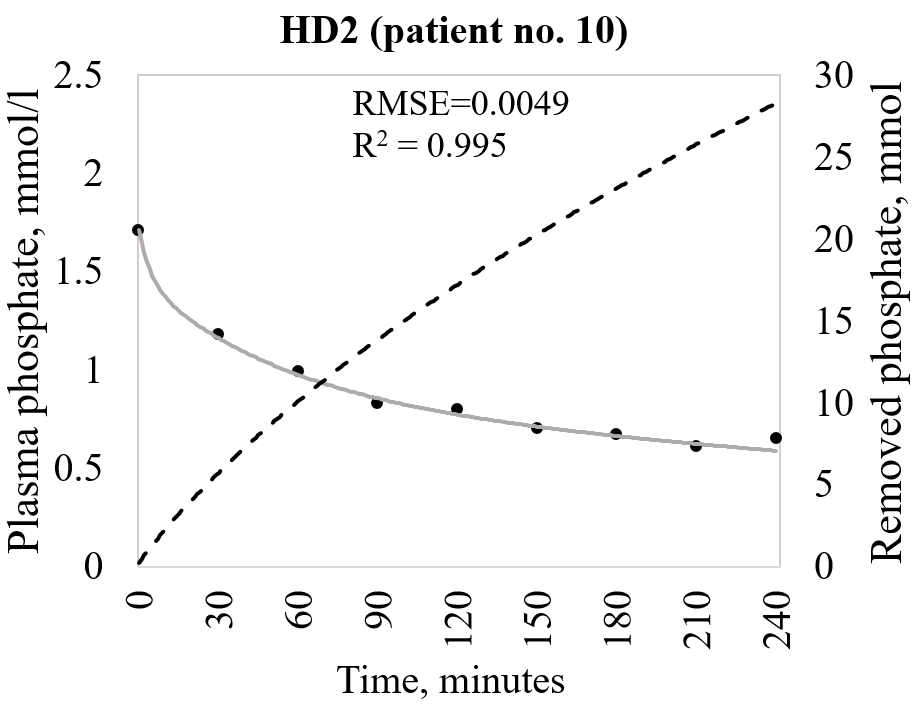


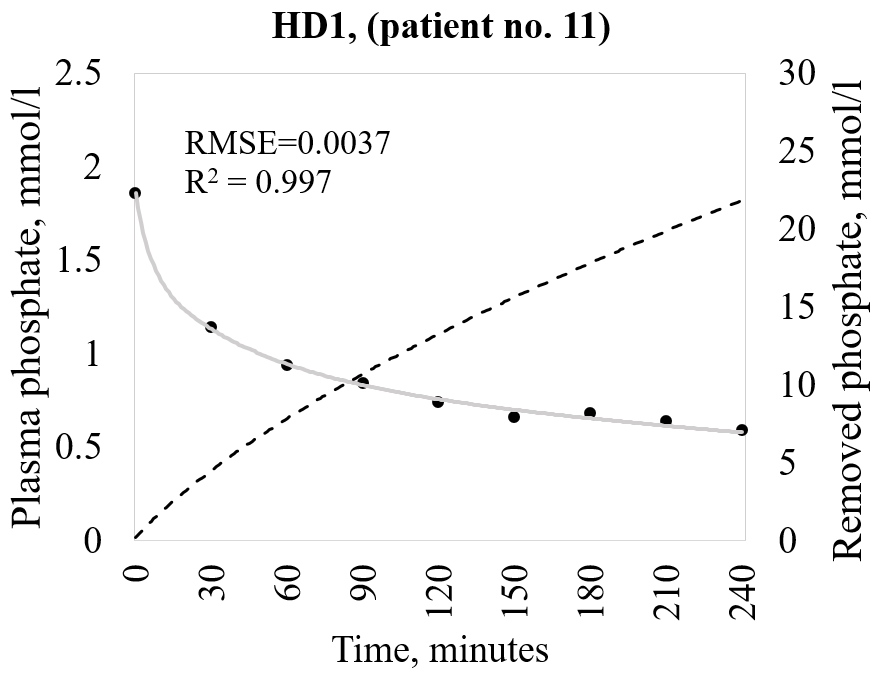

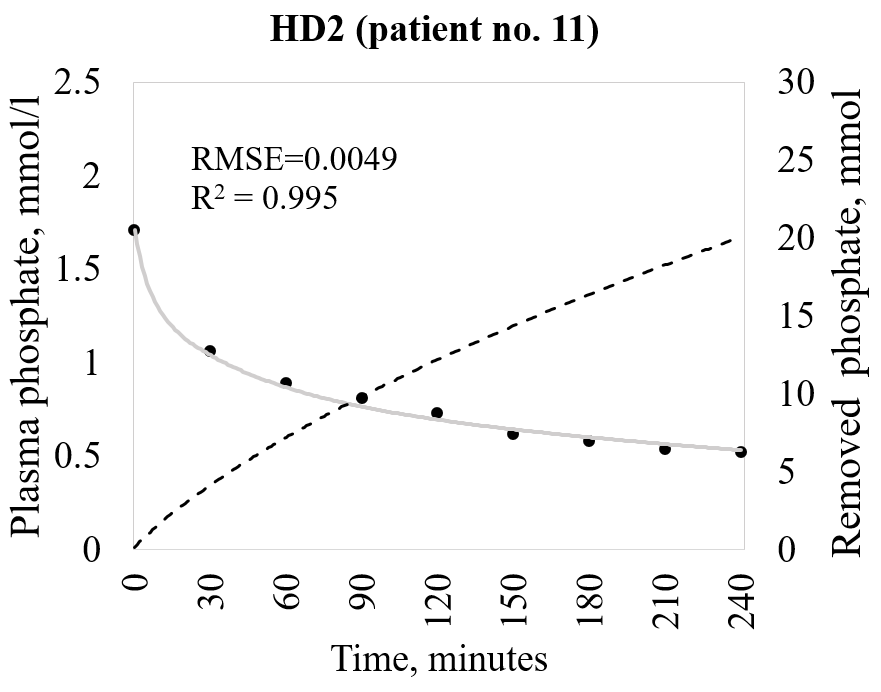


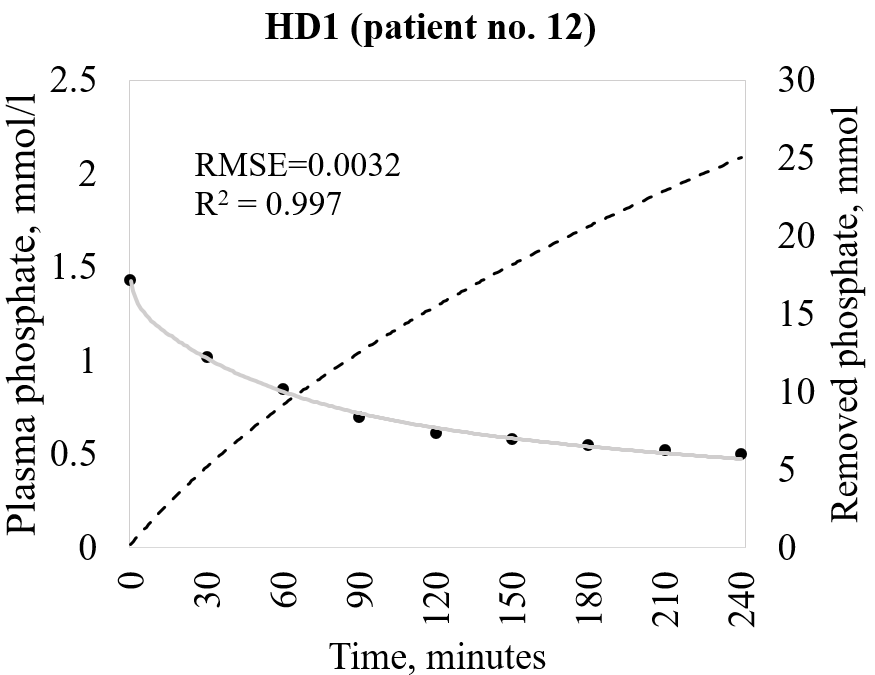

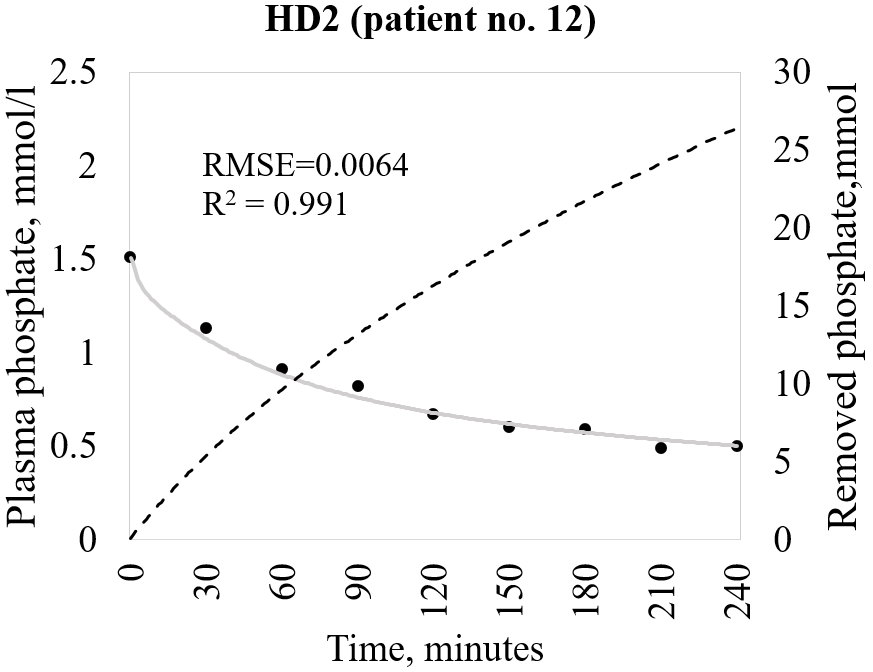

Supplement: Supplementary file 1 — Data S1. [file PHY2-11-e15899-s001.docx]
